# Brazilian Dialysis Survey 2022

**DOI:** 10.1590/2175-8239-JBN-2023-0062en

**Published:** 2023-12-08

**Authors:** Fabiana Baggio Nerbass, Helbert do Nascimento Lima, José Andrade Moura-Neto, Jocemir Ronaldo Lugon, Ricardo Sesso

**Affiliations:** 1 Fundação Pró-Rim, Joinville, SC, Brazil.; 2 Universidade da Região de Joinville, Joinville, SC, Brazil.; 3 Escola Bahiana de Medicina e Saúde Pública, Salvador, BA, Brazil.; 4 Universidade Federal Fluminense, Niterói, RJ, Brazil.; 5 Universidade Federal de São Paulo, São Paulo, SP, Brazil.

**Keywords:** Renal Dialysis, Peritoneal Dialysis, Epidemiology, Diálise Renal, Diálise Peritoneal, Epidemiologia

## Abstract

**Introduction:**

The Brazilian Dialysis Survey (BDS) is an annual national survey about patients on chronic dialysis that contributes to health policies.

**Objective:**

To report the 2022 epidemiological data from the BDS of the Brazilian Society of Nephrology (BSN).

**Methods:**

A survey was carried out in Brazilian chronic dialysis centers using an online questionnaire that included clinical and epidemiological aspects of patients on chronic dialysis, dialysis therapy data, and dialysis center characteristics.

**Results:**

Overall, 28% (n = 243) of the centers answered the questionnaire. In July 2022, the estimated total number of patients on dialysis was 153,831. The estimated prevalence and incidence rates of patients per million population (pmp) were 758 and 214, respectively. Of the prevalent patients, 95.3% were on hemodialysis (HD, 4.6% of these on hemodiafiltration) and 4.7% on peritoneal dialysis (PD). Only 1.3% of patients were not vaccinated against COVID-19. The prevalence of anemia (Hb < 10g/dL) was 27% and hyperphosphatemia (P > 5.5mg/dL) reached 30%. The estimated overall crude annual mortality rate was 17.1%.

**Conclusions:**

The absolute number and prevalence rate of patients on chronic dialysis continue to increase. A growing number of patients were receiving hemodiafiltration. The mortality rate decreased, probably due to the end of COVID-19 pandemic. The conclusions were drawn in the context of relatively low voluntary participation, which imposed methodological limitations on our estimates.

## Resumo

**Introdução:**

O Censo Brasileiro de Diálise (CBD) é uma pesquisa nacional anual sobre pacientes em diálise crônica que contribui para as políticas de saúde.

**Objetivo:**

Informar os dados epidemiológicos de 2022 do CBD da Sociedade Brasileira de Nefrologia (SBN).

**Métodos:**

Foi realizada uma pesquisa em centros brasileiros de diálise por meio de um questionário online que incluiu aspectos clínicos e epidemiológicos de pacientes em diálise crônica, dados da terapia dialítica e características do centro de diálise.

**Resultados:**

No total, 28% (n = 243) dos centros de diálise ativos cadastrados na SBN responderam ao questionário. Em julho de 2022, o número total estimado de pacientes em diálise era de 153.831. As taxas estimadas de prevalência e incidência de pacientes por milhão (ppm) de habitantes foram 758 e 214, respectivamente. Dos pacientes prevalentes, 95,3% estavam em hemodiálise (HD; 4,6% desses em hemodiafiltração) e 4,7% em diálise peritoneal (DP). Apenas 1,3% dos pacientes não foram vacinados contra a COVID-19. A prevalência de anemia (Hb < 10g/dL) foi de 27% e de hiperfosfatemia (P > 5,5mg/dL) alcançou 30%. A taxa bruta total anual de mortalidade estimada foi de 17,1%.

**Conclusões:**

O número absoluto e a taxa de prevalência de pacientes em diálise crônica continuam a aumentar. Um número crescente de pacientes estava em hemodiafiltração. A taxa de mortalidade diminuiu, provavelmente devido ao fim da pandemia da COVID-19. As conclusões foram de um contexto de participação voluntária relativamente baixa, o que impõe limitações metodológicas às nossas estimativas.

## Introduction

Every year, the Brazilian Society of Nephrology (BSN) conducts a national online survey to collect and analyze trends in epidemiological and clinical aspects of patients undergoing chronic dialysis: the Brazilian Dialysis Survey (BDS). In the last decades, this initiative has provided relevant information for the development of health policies and strategies aimed at improving the care of thousands of individuals undergoing chronic dialysis treatment in our country.

In this manuscript, we report the main results of the 2022 BDS.

## Methods

### Data Collection

Dialysis center managers filled out an online questionnaire available on the BSN website. It contained questions about the sociodemographic, clinical, and therapeutic parameters of patients on chronic dialysis and was available from August 2022 to January 2023. Participation in the survey was voluntary, and all dialysis centers registered at BSN were invited to participate by email and BSN media. After the initial invitation, new reminders were sent monthly to centers that had not provided their data. In addition, during the survey period, BSN regional presidents were asked to contact the dialysis centers in their states to reinforce the importance of participation.

### Data Analysis

Data for each center were grouped rather than reported individually. The sample was expanded for national estimates of total number of patients and prevalence rate. We considered that centers that did not answer the questionnaire had the same number of patients as participating centers (mean of 176.4 patients per unit). As this extrapolation may be imprecise, we applied a deviation of ± 2 standard errors of the mean (± 14.1) to account for this estimate in the non-participating centers. Likewise, the mean number of new patients per center was applied to the centers that did not report incidence rates. The results of the sample expansion must be interpreted with caution given that the centers studied were not randomly selected. All other sociodemographic data and patient characteristics relate to the sample studied. The annual mortality and annual incidence of patients on dialysis were estimated from the occurrences of July 2022. National and regional population data from the Brazilian Institute of Geography and Statistics (IBGE) were used to calculate the prevalence and incidence rates. According to this institute, the Brazilian population was 203.06 million inhabitants on August 1^st^, 2022^1^. Most data were descriptive, and the results were compared with previous years.

### Calculations Performed for Estimates

The main calculations and estimates are shown in Table 1.

**Table 1:** Calculations of incidence, prevalence, and mortality estimates

Estimates	Formula
Estimated total number (N) of patients on 1^st^ of July	N of patients in the sample/proportion of participating centers
Estimated annual prevalence rate of dialysis patients (pmp)	Estimated total N of patients on 1^st^ of July/Brazilian population on 1^st^ of August^1^
Estimated total N of patients starting treatment	N of individuals starting treatment in July x 12/proportion of active participating centers
Estimated annual incidence rate of dialysis patients (pmp)	Estimated total N of patients starting treatment/Brazilian population on 1^st^ of August^1^
Estimated total annual N of deaths	N of deaths reported in July x 12/proportion of active participating centers
Estimated crude annual mortality rate (%)	Estimated total N of deaths in 2022 x 100/Estimated N of dialysis patients on 1^st^ of July

pmp: per million population.

## Results

### Estimated Incidence, Prevalence, and Mortality Rates

In July 2022, 872 active chronic dialysis centers were registered at BSN, a 2.7% increase from 2021. In the whole country, there were four dialysis centers per million population (pmp), with lower rates in the Northeast (3.1 pmp) and North (3.3 pmp) regions compared with the Southeast (4.9 pmp), Central-West (5.0 pmp), and South (5.1 pmp) regions.

There were 243 participating centers (28%), a slightly lower percentage than in the previous year (30%). When considering the response rate by number of dialysis centers per region, the region with the highest participation was the South (31%), followed by the Southeast (28%), with the remaining regions having a participation rate of 26%. The number of patients in the current BDS was 2.6% lower than in 2021 (42,868 vs. 44,037).

**Figure 1:**
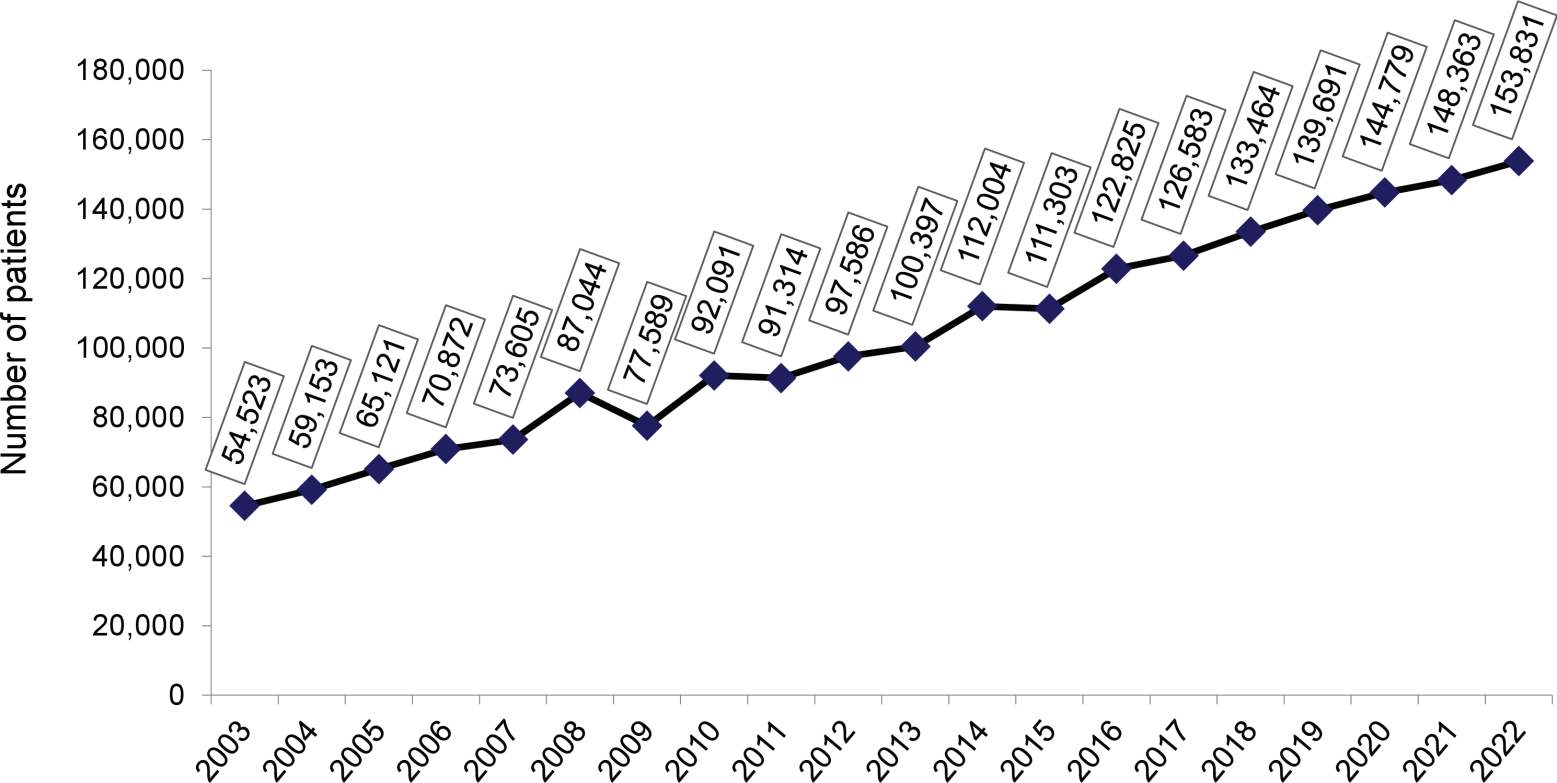
Estimated number of patients on chronic dialysis per year.

**Figure 2:**
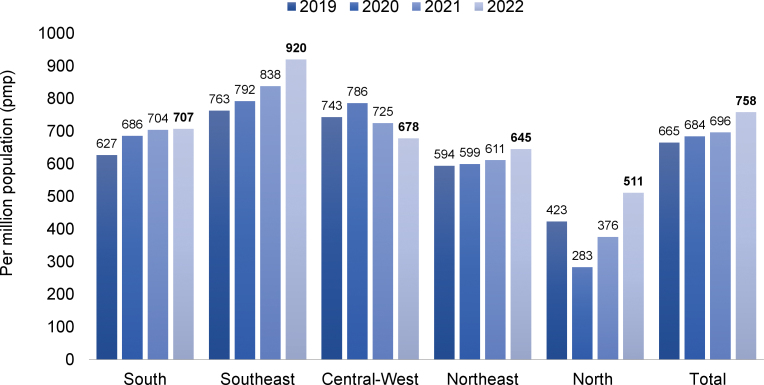
Estimated prevalence rate of patients on dialysis by geographic region in Brazil, per million population.

**Figure 3:**
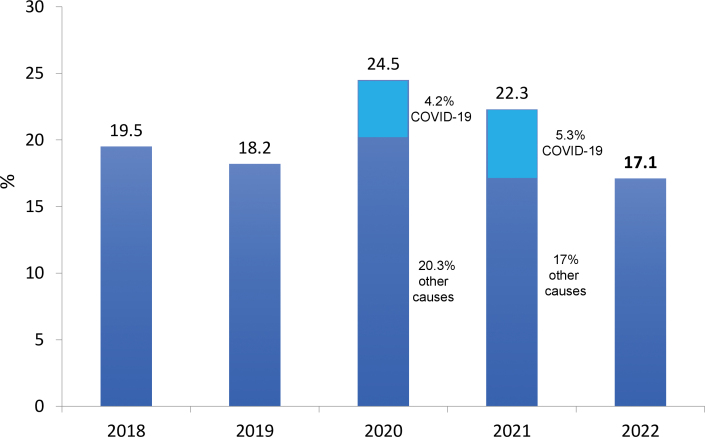
Estimated annual crude mortality rate of dialysis patients.

The estimated total number of patients in July 2022 was 153,831 (variation of ± 2 standard errors = 144,954 to 162,708), 3.7% higher than in July 2021, confirming the trend toward an increase in the number of patients on dialysis observed in recent years (Figure 1). The prevalence rate of dialysis patients also continued to rise, from 696 pmp in 2021 to 758. When examining this indicator by region, a decrease was observed only in the Central-West region (Figure 2). The estimated number of new dialysis patients in 2022 was 43,524. The overall incidence rate was 214 pmp, lower than in 2021, when it reached 224 pmp, ranging from 152 pmp in the North to 269 pmp in the South. The estimated number of deaths for the entire year was 26,929, and the annual crude mortality rate decreased significantly from 22.3% in 2021 to 17.1% in 2022 (Figure 3).

#### Demographic and Clinical Characteristics

**Figure 4:**
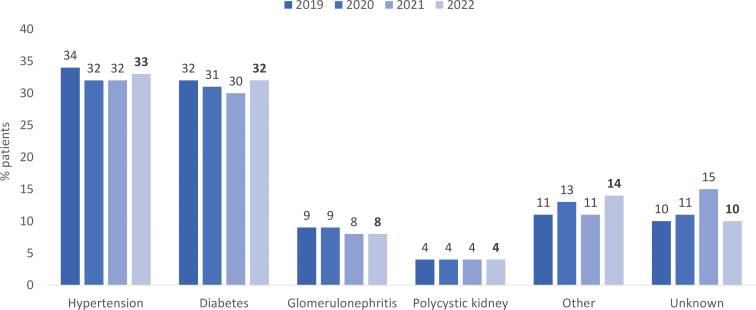
Distribution of dialysis patients according to chronic kidney disease etiology.

**Figure 5:**
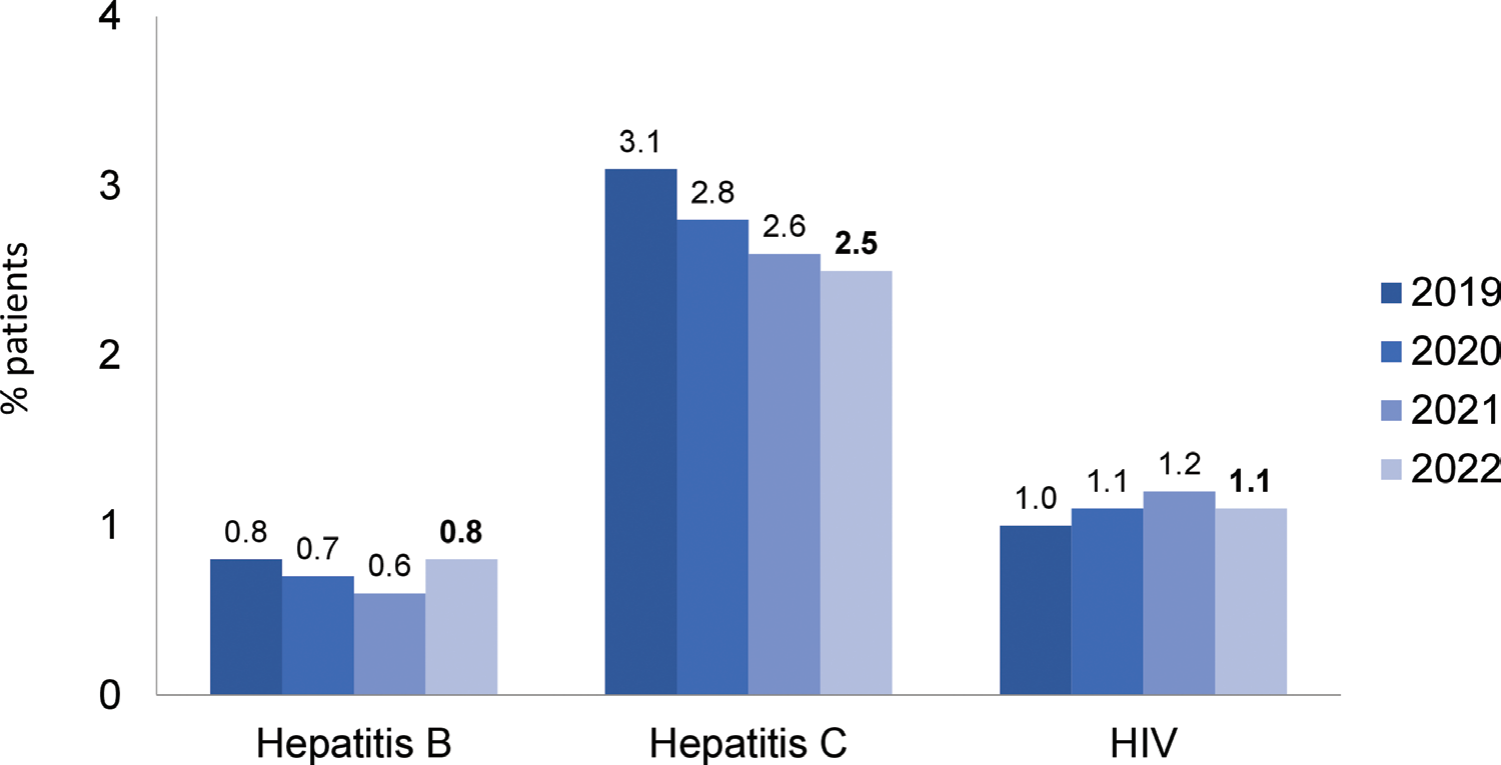
Prevalence of patients with positive serology for hepatitis B and C and HIV.

Together, systemic arterial hypertension (33%) and diabetes mellitus (32%) represented two-thirds of the underlying diseases that led to kidney failure (Figure 4). The distribution of primary diagnosis by region is depicted in the Table S1; “unknown diagnosis” was more common in the North and less frequent in the Southeast region. The percentage of patients with positive serology tests for hepatitis C (2.5%; n = 1,057/42,868) and HIV (1.1%; n = 486/42,868) declined, while that for patients with hepatitis B increased slightly (0.8%; n = 322/42,868) (Figure 5). Only 1.3% (n = 549/42,868) of patients were not vaccinated against COVID-19.

**Figure 6:**
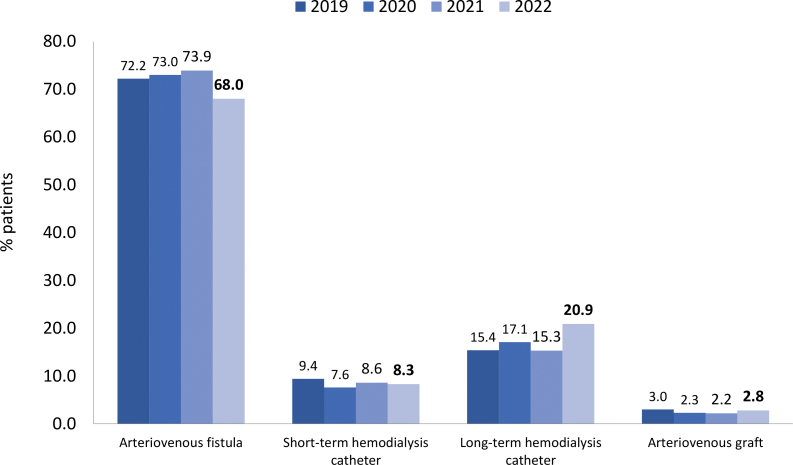
Type of vascular access used for hemodialysis.

**Figure 7:**
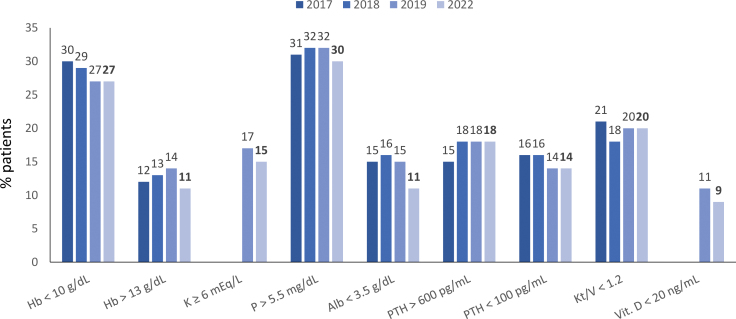
Distribution of dialysis patients according to biochemical results.

Regarding vascular access for HD, 29% (n = 11,967/40,867) of patients used a central venous catheter. The use of long-term catheters increased compared to the previous BDS, accompanied by an equivalent decrease in arteriovenous fistula use (Figure 6). Regarding biochemical parameters, a slight decrease in the prevalence of levels of hemoglobin > 13 g/dL, serum potassium ≥ 6.0 mEq/L, serum phosphate >5.5 mg/dL, serum albumin <3.5 mg/dL, and vitamin D < 20 ng/mL compared to the last time these parameters were included in the survey (2019) (Figure 7).

#### Characteristics of Dialysis Treatment

**Figure 8:**
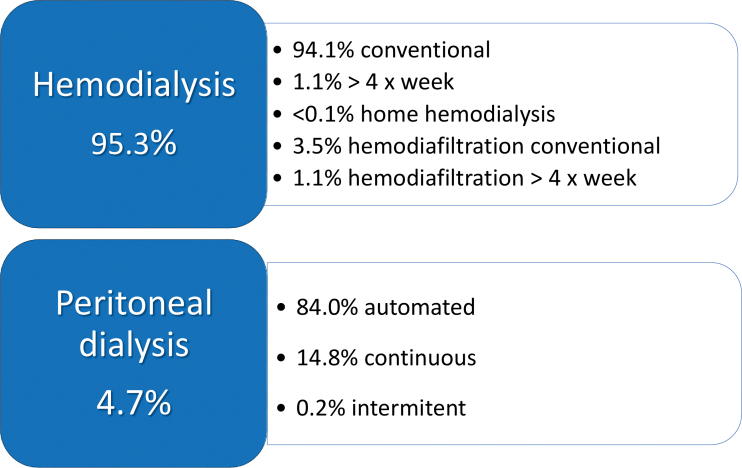
Distribution of patients according to dialysis modality.

The distribution of patients according to dialysis modality and funding source is shown in Table 2 and Figure 8, respectively. The public health system was the funding source for 80.3% (n = 34,420/42,868) and private health insurance for 19.7% (n = 8,448/42,868). Public funding varied across regions, with participation highest in the Northeast (88%), followed by the South (82%), Southeast (80%), North (76%), and Central-West (60%).

**Table 2:** Distribution of patients by dialysis modality and funding source

Modality	Public health		Private health		Total	
	N	%	N	%	N	%
HD ≤ 4 sessions/week	32,901	95.6	5,595	66.2	38,496	89.8
HD > 4 sessions/week	85	0.2	370	4.4	455	1.1
Home HD	0	0.0	25	0.3	25	0.1
HDF ≤ 4 sessions/week	16	0.0	1,439	17.0	1,455	3.4
HDF > 4 sessions/week	5	0.0	431	5.1	436	1.0
CAPD	248	0.7	48	0.6	296	0.7
APD	1,146	3.3	531	6.3	1,677	3.9
IPD	19	0.1	9	0.1	28	0.1
**Total**	34,420	100	8,448	100	42,868	100

HD: hemodialysis; HDF: hemodiafiltration; CAPD: continuous ambulatory peritoneal dialysis; APD: automated peritoneal dialysis; IPD: intermittent peritoneal dialysis.

Regarding dialysis modality, HD was the most common treatment and increased from 94.2 in 2021 to 95.3% in 2022. Among the HD patients, the vast majority were on conventional HD (94.1%; n = 38,496/40,867), 1.1 % (n = 455/40,867) were on HD > 4x/week, and less than 0.1% (n = 25/40,867) were on home HD. There was an increase in the prevalence of patients on hemodiafiltration, from 1.8 in 2021 to 4.6% (n = 1,891/40,867) in 2022. Of the 4.7% (n = 2,001/42,868) of patients on peritoneal dialysis (PD), 84% (n = 1,677/2,001) were on automated peritoneal dialysis (APD).

#### Characteristics of Participating Centers

Of the 243 participating dialysis centers, 75.7% were privately owned, 15.6% were philanthropic, and 8.6% were public. Most centers were identified as satellite centers (61.7%), and in-hospital centers comprised 38.3%. The national mean number of patients per nephrologist, nurse, and nurse technician was 30, 39, and 7, respectively.

## Discussion

In this study, we described the main results of the BDS 2022. For most variables, the trends observed in recent years were maintained. Exceptions included a significant increase in the prevalence of patients on hemodiafiltration as dialysis modality, an increase in those using long-term central venous catheters, and a decrease in the crude mortality rate.

The participation rate of dialysis centers in 2022 slightly decreased compared to the previous year, from 30% to 28%^2^. The upward trend in the total number of patients by 3.7% and the prevalence by 2.8 followed the pattern observed over time.

The overall prevalence of people on dialysis (758 pmp) was higher than the average of the 2019 Latin American Society of Nephrology (SLAHN) registry (650 pmp)^3^ and the 2020 European registry (587 pmp)^4^. On the other hand, our numbers were substantially lower than those from the United States in 2019 (1,696 pmp)^5^. The 2022 incidence rate (214 pmp) was slightly lower than the 2021 national estimate (224 pmp), higher than the Latin American estimate for 2019 (168 pmp)^3^ and the European estimate for 2020 (125 pmp)^4^ and lower than the United States estimate for 2020 (372 pmp)^5^.

There was a significant decrease in crude mortality rate, from 22.3% in 2021 to 17.1% in 2022. The 2022 rate is slightly lower than the rates observed in the years before the COVID-19 pandemic, around 18 to 20%. Considering that the mortality and incidence rates have decreased, the observed increase in prevalence may reflect better care for patients on dialysis.

Hypertension and diabetes were the main primary causes of chronic kidney disease and had similar rates, 33 and 32%, respectively.

The percentage of patients with hepatitis C continued to decrease, reaching 2.5% for the first time. A minority of patients were not vaccinated against COVID-19, probably due to their own decision, as vaccination was widely available to the population in this period.

For the first time, the percentage of patients using long-term central venous catheters surpassed one-fifth of all hemodialysis patients (20.9%); it was 15.3% in 2021 and 6.0% ten years ago (2013)^6^. The prevalence of arteriovenous fistula use decreased significantly in the last year (73.9 to 68%), whereas prevalence of short-term central venous catheter and arteriovenous graft were similar. The reasons for this increase in catheter use require confirmation through additional epidemiological studies and further investigation, as data was collected from a voluntary sample of centers. The experience that nephrologists have gained in recent years implanting long-term catheters may have contributed to this finding. In contrast, barriers to increased use of native vascular access (arteriovenous fistula) persist, highlighting the need for efforts to facilitate its creation and use.

For the second time, the survey assessed the percentage of patients on hemodiafiltration (HDF), and found an increase from 1.8% to 4.6% of all patients on hemodialysis (from 2021 to 2022, respectively). Further investigation is warranted to determine whether this is unique to the centers that submitted data or a national trend. The latter rate is still below, but closer to, the global prevalence, estimated at 10% in 2018^7^.

Anemia and hyperphosphatemia remained the most prevalent biochemical disorders, showing that the current treatments available are not effective for all patients.

The prevalence of patients on peritoneal dialysis continued to decrease, accounting for only 4.7%, although almost half of the participating centers (47%) offer PD as a treatment option. The model proposed by our public health system, which is not economically viable for most clinics, seems to be the main reason^8^.

As study limitations, we highlight the electronic data collection through voluntary participation, the grouping of patient data by dialysis center, and the lack of validation of the answers. Furthermore, due to the non-random inclusion of approximately 28% of active dialysis centers, the estimates of national prevalence and incidence rates have limited accuracy. Caution is advisable for data interpretation. It cannot be ruled out that the non-participating centers were smaller, less well-organized facilities that had no interest in statistics or were hesitant to disclose less favorable outcomes. These potential biases may have introduced uncertainties into our estimates.

In conclusion, despite the limitations of the study, the BDS 2022 confirmed a consistent increase in the prevalence of dialysis patients over the years and identified a growing number of patients receiving hemodiafiltration. The mortality rate decreased to figures similar to the pre-COVID-19 pandemic period.

## Supplementary Material

The following online material is available for this article:

Table S1 – Distribution of dialysis patients according to chronic kidney disease etiology across geographic regions.
