# Supplementary material for: Brazilian Dialysis Survey 2022
Source: J Bras Nefrol. 2023 Dec 8;46(2):e20230062. doi: 10.1590/2175-8239-JBN-2023-0062en (PMC11210532; doi:10.1590/2175-8239-JBN-2023-0062en)
Supplement: Supplementary file 1 [file 2175-8239-jbn-2023-0062-s1.pdf]

Braz. J. Nephrol. (São Paulo)

https://doi.org/10.1590/2175-8239-JBN-2023-0062en

Supplementary Material to "Brazilian Dialysis Survey 2022 "

Table S1. Distribution of dialysis patients according to chronic kidney disease etiology across geographic regions.

Region	Hypertension	Diabetes	Glomerulo-nephritis	Polycystic kidney	Other	Unknown
Central-West (%)	33.0	33.1	10.6	4.4	7.9	11.0
Northeast (%)	36.4	30.2	6.9	2.9	12.5	11.1
North (%)	21.3	31.4	2.9	2.7	16.3	25.4
Southeast (%)	36.0	33.4	8.9	4.3	8.6	8.8
South (%)	26.1	32.1	8.8	5.4	15.9	11.7
